# Effect of 15° Reverse Trendelenburg Position on Arterial Oxygen Tension during Isoflurane Anesthesia in Horses

**DOI:** 10.3390/ani12030353

**Published:** 2022-02-01

**Authors:** Laura Tucker, Daniel Almeida, Erin Wendt-Hornickle, Caroline F. Baldo, Sandra Allweiler, Alonso G. P. Guedes

**Affiliations:** 1 Veterinary Clinical Sciences Department, College of Veterinary Medicine, University of Minnesota, St. Paul, MN 55108, USA; tuckerl@uoguelph.ca (L.T.); alme0061@umn.edu (D.A.); ewendtho@umn.edu (E.W.-H.); Caroline.Baldo@bsci.com (C.F.B.); Sandra_Allweiler@hotmail.com (S.A.); 2 Department of Clinical Studies, Ontario Veterinary College, University of Guelph, Guelph, ON N1G 2W1, Canada; 3 Boston Scientific, Research and Technology Center, Arden Hills, MN 55112, USA

**Keywords:** hypoxemia, intrapulmonary shunt, complication, ventilation, junctional escape-capture bigeminy, general anesthesia, dorsal recumbency

## Abstract

**Simple Summary:**

Horses commonly develop low blood oxygen levels during anesthesia, especially when they are placed on their backs. This study investigated whether a 15° head-up tilt, in a homogenous group of anesthetized horses positioned on their backs, would result in better blood oxygen levels as compared to no tilt. The results showed significantly greater blood oxygen levels with tilt compared to no tilt in five out of six horses tested. In one horse the effect was the opposite. The concurrent effect on cardiovascular function remains to be tested in detail. Further studies are needed to confirm these findings in a larger group of horses and to determine the effects on blood pressure and treatment options.

**Abstract:**

Lower than expected arterial oxygen tension (PaO_2_) continues to be an unresolved problem in equine anesthesia. The aim of this randomized, crossover, and prospective study using six adult horses is to determine if a 15° reverse Trendelenburg position (RTP) increases PaO_2_ during inhalation anesthesia. Under constant-dose isoflurane anesthesia, dorsally recumbent horses were positioned either horizontally (HP) or in a 15° RTP for 2 h. Lungs were mechanically ventilated (15 mL/kg, 6 breaths/min). Arterial carbon dioxide tension (PaCO_2_), PaO_2_, inspired oxygen fraction (FiO_2_), and end-tidal carbon dioxide tension (EtCO_2_) were determined every 30 min during anesthesia. Indices of dead-space ventilation (Vd/Vt), oxygenation (P–F ratio), and perfusion (F–shunt) were calculated. Dobutamine and phenylephrine were used to support mean arterial pressure (MAP). Data are presented as median and range. In one horse, which was deemed an outlier due to its thoracic dimensions and body conformation, indices of oxygenation worsened in RTP compared to HP (median PaO_2_ 438 vs. 568 mmHg; P–F ratio 454 vs. 586 mmHg, and F–shunt 13.0 vs. 5.7 mmHg). This horse was excluded from calculations. In the remaining five horses they were significantly better with RTP compared to HP. Results in remaining five horses showed that PaO_2_ (502, 467–575 vs. 437, 395–445 mmHg), P-F ratio (518, 484–598 vs. 455, 407–458 mmHg), and F-shunt (10.1, 4.2–11.7 vs. 14.2, 13.8–16.0 mmHg) were significantly different between RTP and HP (*p* = 0.03). Other variables were not significantly different. In conclusion, the 15° RTP resulted in better oxygenation than HP in dorsally recumbent, isoflurane-anesthetized horses, although worsening of oxygenation may occur in individual horses. A study detailing the cardiovascular consequences of RTP is necessary before it can be recommended for clinical practice.

## 1. Introduction

Respiratory function impairment, or less than expected arterial partial pressure of oxygen (PaO_2_), has long been recognized and continues to be very common in equine anesthesia [1,2,3,4,5,6,7,8]. Horses in dorsal recumbency appear to be particularly prone to impaired oxygenation, although it can also occur in lateral recumbency [5,9]. The problem arises primarily from increased intrapulmonary right-to-left shunting, secondary to the development of alveolar atelectasis in dependent lung areas [5,10]. Compression atelectasis appears to be the most common [1,5,10], although absorption atelectasis might also occur in some situations [6]. Physical features such as body mass, chest dimensions, and abdominal shape all have been shown to positively correlate with the degree of impairment [1,10,11,12]. 

Several physical and pharmacological interventions aimed at mitigating impaired oxygenation have been studied in anesthetized horses. These include reverse Trendelenburg position (RTP) [13,14], positive end-expired pressure (PEEP) combined with repeated alveolar recruitment maneuvers (RM) [15,16], continuous positive airway pressure [17], intravenous and inhaled β-adrenergic receptor agonists [18,19], inhaled nitric oxide [10,20,21], acepromazine [7], and reducing FiO_2_ [22,23,24,25]. Overall, physical methods appear to improve PaO_2_ more consistently than pharmacological methods, which seems logical given the prominent contribution of compression atelectasis to impaired oxygenation [5,10]. 

In morbidly obese human patients, the reverse Trendelenburg position (RTP; i.e., head-up tilt) was initially advocated to facilitate upper abdominal surgery [26]. A 30° RTP was subsequently shown to be efficacious in improving pulmonary gas exchange and total respiratory system compliance [27,28,29]. In isoflurane-anesthetized horses [13,14] and sevoflurane-anesthetized steers [30] positioned in dorsal recumbency, RTP at angles between 5° and 10° did not improve arterial oxygenation compared to the horizontal position (HP). Another study comparing 7° RTP with a 7° head-down (i.e., Trendelenburg) position for 90 min in dorsally recumbent isoflurane-anesthetized horses breathing 0.85 FiO_2_ found that the RTP did not improve arterial oxygenation compared to baseline. However, gas exchange was better maintained compared to the head-down position [31]. Finally, a recent clinical study employing 5° RTP did not show any significant improvement in arterial oxygen tension in isoflurane-anesthetized horses positioned in dorsal or lateral recumbency [13]. Thus, these veterinary studies using relatively conservative RTP angles compared to that commonly applied in humans do not appear to support the use of RTP to improve gas exchange compared to the HP during inhalation anesthesia in the horse. However, it remains unclear if steeper RTP angles would result in improved arterial oxygen tension. 

The study aimed to determine the effect of a 15° RTP on arterial oxygen tension in dorsally recumbent, mechanically ventilated, and isoflurane-anesthetized horses. It was hypothesized that arterial oxygen tension would be significantly better with a 15° RTP as compared to the HP. Effects on the alveolar dead-space ventilation (Vd/Vt) and the oxygen content-based index (F–shunt) were evaluated as secondary goals. Results indicated that a 15° RTP resulted in better PaO_2_ than HP in dorsally recumbent, isoflurane-anesthetized horses, but may lead to negative cardiovascular consequences that warrant further investigations.

## 2. Materials and Methods

### 2.1. Animals and Study Design

A total of 6 horses (1 mare and 5 geldings) aged 8.5 ± 3 years (range 5–13 years) of several breeds (1 Arabian, 3 Quarter Horses, and 2 mixed breeds) and weighing 462 ± 50 kg (range 397–536 kg) were studied in a randomized (randomization.com; accessed on 3 July 2017) crossover design with two treatments, separated by at least one-week washout in between. Horses were deemed healthy based on physical exam, complete blood cell counts, and serum biochemistry analyses. 

### 2.2. Anesthesia

Food was withheld for approximately 12 h and water was available *ad libitum* prior to anesthesia. Thoracic dimensions were obtained as previously described [12] and horses were classified subjectively as round-bellied or flat-bellied, according to their abdominal contour as described by Moens and co-workers [11]. A 14-gauge intravenous catheter (Angiocath)^a^ was aseptically placed in one jugular vein for drug and fluid administration. Horses were sedated with 5 μg/kg dexmedetomidine (Dexdomitor; Orion Pharma, Orion Corporation, Espoo, Finland) or 1 mg/kg xylazine (AnaSed; Akorn, Inc., Lake Forest, IL, USA) as part of a separate study [32]. After 5–7 min, anesthesia was induced with 0.06 mg/kg midazolam (Midazolam Injection USP; Hospira Inc., IL, USA) and 2.2 mg/kg ketamine (KetaVed; Vedco Inc., Saint Joseph, MO, USA) behind a swing gate and inside of a padded stall. The horse’s trachea was orally intubated with a 26 mm internal diameter cuffed endotracheal tube. The horse was subsequently hoisted and positioned in dorsal recumbency on a padded table (see positioning description below) and connected to a large animal circle breathing system and anesthesia machine (Mallard; Mallard Medical Inc., Redding, CA, USA). General anesthesia was maintained with an end-tidal isoflurane (IsoFlo; Abbott Animal Health, Abbot Park, IL, USA) concentration of 1.2 times its minimum alveolar concentration (MAC), considered to be 1.31% [33], with an oxygen flow rate of at least 6l/min. The target end-tidal isoflurane concentration was achieved in all horses within 10 min after connecting the endotracheal tube to the breathing circuit of the anesthesia machine. Horses received fluid therapy with Hartmann’s solution (Vetivex; Dechra Veterinary Products, Overland Park, KS, USA) at 3 mL/kg/h.

### 2.3. Body Positioning Protocols during Anesthesia

**Figure 1:**
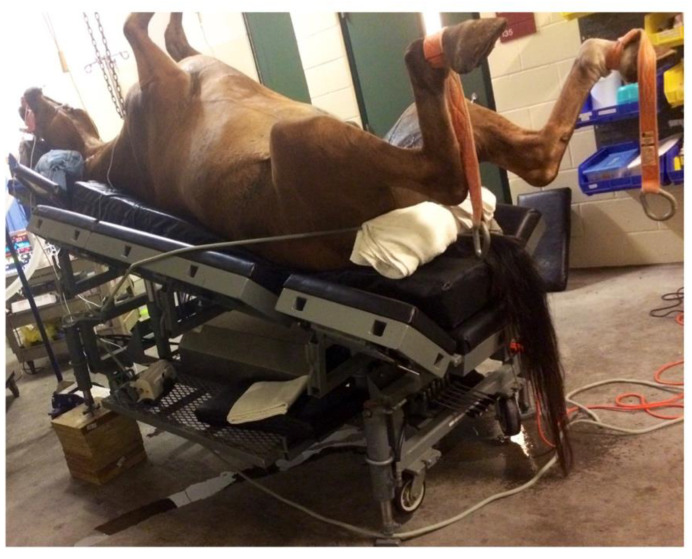
Isoflurane-anesthetized horse positioned in 15° reverse Trendelenburg. To prevent the possibility of horses sliding off the table, a rope padded with towels was placed around the horse’s hindquarters, approximately at the level of the ischiatic tuberosity, with each end secured to the head of the table.

The anesthetized horse was hoisted onto a surgical operating table capable of hydraulic tilting, which was previously adjusted to either the HP or with a 15° RTP. The angle of the table was measured using a 25.4 cm multi-function standard digital level (Husky; The Home Depot, Atlanta, GA, USA) that was positioned on a fixed solid part of the table. To prevent the possibility of horses sliding off the table, a rope padded with towels was positioned around the horse’s hindquarters, approximately at the level of the ischiatic tuberosity, with each end secured to the head of the table (Figure 1). Horses were maintained in the designated position for 2 h.

### 2.4. Monitoring and Support during Anesthesia

Horses were monitored with base-apex electrocardiography. A 20-gauge, 4.4 cm catheter (Insight)^a^ was aseptically placed in the facial artery and connected via a fluid-filled regular 83.3 cm extension set to a calibrated disposable pressure transducer (BD DTX^TM^ disposable pressure transducers; Becton Dickinson Infusion Therapy Systems, Sandy, UT, USA) that was zeroed and positioned at the level of the heart (i.e., scapulohumeral joint) for invasive blood pressure measurement. Dobutamine (DOBUTamine injection USP; Hospira Inc., Lake Forest, IL, USA) was administered as a continuous infusion as needed to maintain mean arterial pressure (MAP) between 70 and 80 mmHg. A dobutamine dose was started at 0.5 μg/kg/min and then doubled every 2–3 min until MAP was within the desired range. If signs of dobutamine-induced side effects occurred with a given dose (tachycardia or cardiac arrhythmias), its dose was reduced to the previous infusion rate and phenylephrine (Neo-Synephrine^®^ HCl; Hospira Inc., Lake Forest, IL, USA) was administered for additional cardiovascular support as 1–2 μg/kg boluses to maintain MAP within the desired range. Approximately 2–3 min was allowed after each bolus before the next one was administered if needed. The amounts of dobutamine and phenylephrine used in both treatment groups were recorded.

The horses’ lungs were mechanically ventilated (Mallard; Mallard Medical Inc., Redding, CA, USA) at a rate of 6 breaths/minute and tidal volume 15 mL/kg, rounded to the nearest liter, with an inspiratory time of 2 s. Peak inspiratory pressures, as measured by the pressure gauge located in the breathing circuit of the anesthesia machine, were recorded. Airway gas samples were obtained continuously throughout anesthesia using a calibrated multi-parameter monitor (DPM 7; Mindray DS USA Inc., Mahway, NJ, USA) for side stream monitoring of inspired and end-tidal concentrations of oxygen, carbon dioxide, and isoflurane. Arterial blood samples (3 mL) were collected at 30, 60, 90, and 120 min of anesthesia for the determination of blood gas tensions, pH, and lactate concentrations using a calibrated portable blood gas analyzer (i-STAT CG4 test cartridges; Abbott Point of Care Inc, NJ, USA). Rectal temperature was monitored with a digital thermometer.

### 2.5. Recovery from Anesthesia

At the end of the experiment, the urinary bladders were emptied via catheterization, monitoring equipment was removed, the orotracheal tube was disconnected from the breathing circuit, and the horses were transferred to padded recovery stalls for unassisted recovery as part of a separate study [32].

### 2.6. Calculations

The PaO_2_ and the ratio between arterial oxygen tension and a fraction of inspired oxygen (P–F ratio) were used to assess oxygenation. The P-F ratio was determined by dividing the arterial oxygen tension (PaO_2_) by the inspired oxygen fraction (FiO_2_). The Vd/Vt was calculated via the Enghoff modification of the Bohr equation where Vd/Vt = ([PaCO_2_ − P_ET_CO_2_]/PaCO_2_) × 100 [34]. The F-shunt was calculated using the equation ([Cc′O_2_ − CaO_2_]/[Cc′O_2_ − CaO_2_ + 3.5 mL/dl]) × 100 [13,14,35,36], where Cc′O_2_ and CaO_2_ were, respectively, the capillary and arterial oxygen contents. The value 3.5 is the arteriovenous oxygen content difference in mechanically ventilated humans [37], which closely approximates that of isoflurane-anesthetized, dorsally recumbent horses [23,38]. The CaO_2_ was calculated with the equation (1.38 × Hb × SaO_2_) + (PaO_2_ × 0.003), where 1.38 is the oxyphoric capacity of equine hemoglobin [39], Hb is the hemoglobin concentration measured in the awake horse before the experiment, SaO_2_ is the arterial hemoglobin saturation with oxygen determined via blood gas analysis, and 0.003 is the oxygen solubility in blood. The Cc′O_2_ was calculated similarly, except that the pulmonary end-capillary partial pressure of oxygen was assumed to be equal to the alveolar oxygen partial pressure (P_A_O_2_), which was estimated using the alveolar gas equation in which P_A_O_2_ = (P_B_ × FiO_2_-P_H2O_) − (PaCO_2_/0.8). The PB is the local barometric pressure on the day and time of the experiment (range 761–766 mmHg), P_H2O_ is the partial pressure of water (47 mmHg), and 0.8 is the carbon dioxide production to oxygen consumption ratio. Total respiratory system plus breathing system compliance (C_tot_) was calculated as peak volume/pressure and was corrected for body weight (mL/cmH_2_O/kg). 

### 2.7. Statistical Analysis

Statistical analyses were performed using commercial software (GraphPad Prism Version 7.0d for MAC OS, GraphPad Software, Inc., San Diego, CA, USA). The data were assessed for normality via visual inspection of QQ plots and the Shapiro–Wilk normality test. Indices of oxygenation (PaO_2_ and P–F ratio) and indices of ventilation and perfusion (PaCO_2_, P_ET_CO_2_, Vd/Vt, F–shunt) over time were analyzed by means of two-way repeated measures ANOVA followed by Holm–Sidak post-test. The median PaO_2_, P–F ratio, F-shunt, and C_tot_ over the 2 h of anesthesia were compared between groups using Wilcoxon matched–pairs signed–rank test. Spearman correlation was used to determine the relationship between changes in PaO_2_ and C_tot_. Statistical significance was set at *p* < 0.05. Data are shown as mean ± SD or median (range).

## 3. Results

All horses completed the study and recovered uneventfully. Demographic information for each horse is presented in Table 1. One horse (#5) was excluded from statistical analysis due to abdominal contour and thoracic length that was markedly different from the remaining five horses.

### 3.1. Respiratory Variables

**Figure 2:**
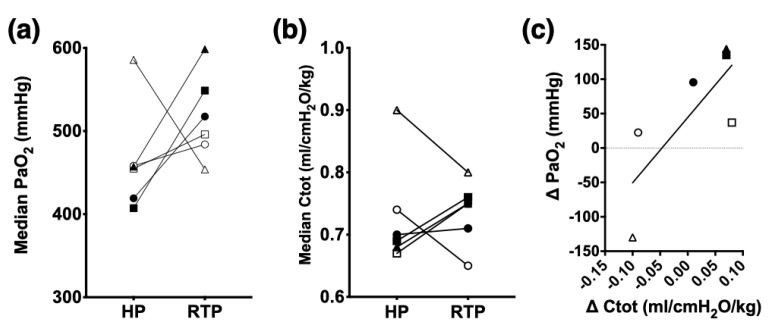
Individual changes in arterial median oxygen tension (PaO_2_; panel **a**), total respiratory system compliance (C_tot_; panel **b**), and Spearman correlation between changes in PaO_2_ and C_tot_ (panel **c**) during 2 h of isoflurane anesthesia in 6 dorsally recumbent, mechanically ventilated horses positioned either horizontally (HP) or in a 15° reverse Trendelenburg position (RTP). Measurements were obtained every 30 min during the 2 h of anesthesia in each designated position. Individual horses are identified with a unique symbol: open square (horse #1), filled square (#2), open circle (#3), filled circle (#4), open triangle (#5), and filled triangle (#6). Horse #5 (open triangle) was considered an outlier and excluded from statistical comparison. Median PaO_2_ during RTP (502 mmHg) and HP (437 mmHg) were significantly different (*p* = 0.03; Wilcoxon matched-pair sign rank test; *n* = 5). Median C_tot_ during HP (0.69 mL/cmH_2_O/kg) and RTP (0.75 mL/cmH_2_O/kg) were not significantly different (P=0.62; Wilcoxon matched–pair sign–rank test; *n* = 5). Spearman correlation between changes (RTP minus HP) in PaO_2_ and C_tot_ was 0.64 with *p* = 0.09 (*n* = 6).

Indices of oxygenation, ventilation, and perfusion are summarized in Table 2. Statistically significant differences were detected between HP and RTP for PaO_2_, P-F ratio, and F-shunt at several time points during anesthesia. The treatment effect accounted for approximately half of the total variance (after adjusting for matching) with <1% chance of randomly observing the measured effect in an experiment of this size. The PaCO_2_ and Vd/Vt were not significantly different between RTP and HP. The Vd/Vt increased over time during RTP such that it was significantly greater at 90 and 120 min compared to 30 min of anesthesia. The 2 h median PaO_2_ of each horse is shown in Figure 2a, and the difference between group medians (437 mmHg and 502 mmHg during HP and RTP, respectively) was statistically significant (*p* = 0.03). The median P-F ratio was significantly (*p* = 0.03) greater during RTP (518 mmHg, range 484–598) than HP (455 mmHg, range 407–458). F-shunt was significantly lower during RTP (10.1%, range 4.2–11.7) than HP (14.2%, range 13.8–16.0). Individual C_tot_ values for each horse during HP (median 0.69 mL/cmH_2_O/kg) and RTP (median 0.75 mL/cmH_2_O/kg) are shown in Figure 2b, and the group medians were not significantly different. Spearman correlation between changes in PaO_2_ and C_tot_ for all horses (*n* = 6) was 0.64 (*p* = 0.09), and the results are shown in Figure 2c.

### 3.2. Clinical Cardiovascular and Other Variables

**Figure 3:**
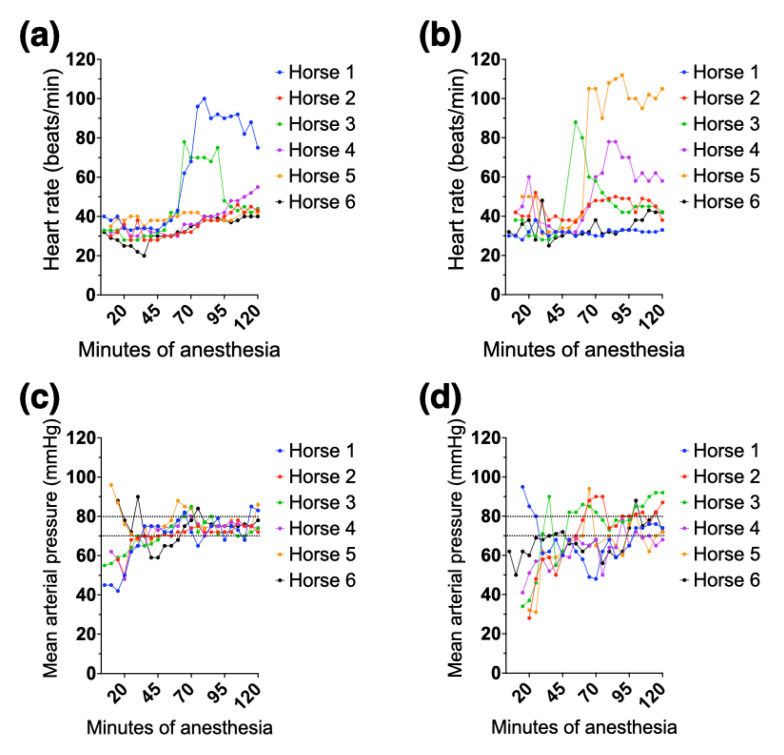
Individual heart rate (top panels) and mean arterial pressure (bottom panels) recorded every 5 min during 2 h of isoflurane anesthesia in 6 healthy adult horses while positioned either horizontally (**a**,**c**) or on a 15° reverse Trendelenburg position (**b**,**d**). The dotted lines in the bottom panels indicate the target range for mean arterial pressure. Horses 5 and 6 in panel (**c**) and horses 1 and 6 in panel (**d**) were already receiving dobutamine at the first recording of mean arterial pressure.

**Figure 4:**
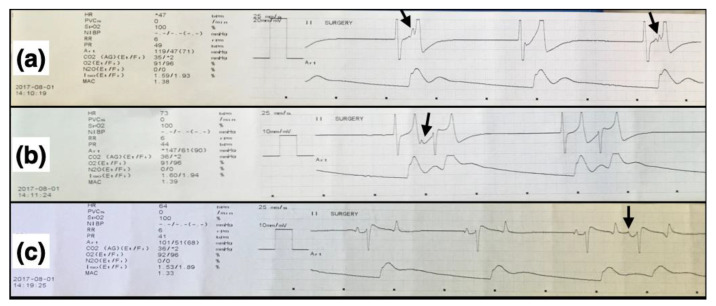
Electrocardiographic (top traces) and arterial blood pressure (bottom traces) recordings in one horizontally positioned, dorsally recumbent isoflurane-anesthetized horse that developed junctional escape-capture bigeminy during dobutamine administration. (**a**) Junctional rhythm with blocked P waves (arrows) that can be seen in the first and third, but not in the second, complexes. (**b**) A junctional escape-capture bigeminy is seen, and a P wave can be seen in the first (arrow) but not in the second set of bigeminal complexes. (**c**) A normal sinus rhythm with occasional atrial premature complexes (arrow), and a base-apex electrocardiogram, recorded at 25 mm/s and either 20 mm/mV (**a**) or 10 mm/mV (**b**,**c**), can be seen.

Median rectal temperature, dobutamine, and phenylephrine use during HP and RTP, as well as the body position during the first anesthesia for each horse, are listed in Table 3. Clinical cardiovascular parameters (HR, MAP), rectal temperature, and requirement of dobutamine and phenylephrine were not compared statistically between groups. The heart rates and mean arterial pressures for individual horses are shown in Figure 3. Cardiac dysrhythmias were observed during both HP and RTP. During HP, dysrhythmias included tachycardia (heart rate > 50 beats/min) in three horses (horses # 1, 3 and 4), atrial premature complexes in one horse (horse #2), and sinus bradycardia (heart rate < 26 beats/min) followed by junctional escape rhythm, junctional escape-capture bigeminy, and atrial premature complexes in one horse (horse #6, shown in Figure 4). During RTP, dysrhythmias included tachycardia in four horses (horses # 2, 3, 4, and 5) and atrial premature complexes in one horse (horse #6).

## 4. Discussion

The results of the current study suggest that a 15° RTP can significantly improve arterial oxygen tension compared to the HP in dorsally recumbent, mechanically ventilated, isoflurane-anesthetized horses. However, worsening of arterial oxygenation can also happen, so its use in clinical cases should be very carefully monitored. The 15° RTP better preserved the V/Q relationship compared to HP by decreasing venous admixture, as indicated by a significantly lower F–shunt. These findings are in agreement with reports in mechanically ventilated morbidly obese humans at 30° RTP [27,28,29] but are in contrast to those obtained in mechanically ventilated horses at 5° RTP [13] or 7° RTP [14] and in spontaneously breathing steers at 5° and 10° RTP [30]. It is important to note that our results were obtained in a homogeneous group of round-bellied horses, a group of horses known to have lower PaO_2,_ and a higher alveolar-to-arterial oxygen tension difference than flat-bellied horses in both dorsal and lateral recumbency [11]. Further studies are needed to confirm or refute our results in round-bellied horses given the relatively small sample size, as well as to determine the response of flat-bellied horses to RTP. Interestingly, the PaO_2_ decreased during RTP in relation to HP in the single horse classified as flat-bellied. It is likely that a proportion of the general equine population will not respond satisfactorily to RTP as is the case with approximately a third of morbidly obese human patients [29].

The actual body mass may also affect the efficacy of RTP in improving PaO_2_. In humans, RTP resulted in significantly better PaO_2_ during anesthesia compared to HP in morbidly obese individuals [27,28] but not in non-obese individuals with similar body mass [40]. In horses, a non-significant trend was reported for PaO_2_ and P-F ratio, which were higher in 7° RTP than in HP in horses weighing between 300 and 599 kg but lower in those weighing 600 kg or more [14]. In morbidly obese human patients, 30° RTP improves PaO_2_ at least in part by increasing total respiratory system compliance [27,28,29]. In the current study, a similar trend was observed in four out of six horses, and a strong positive correlation between changes in PaO_2_, and C_tot_ was observed, albeit these were outside the pre-set statistical significance level. The markedly greater C_tot_ in the single flat-bellied (and long-chested) horse during HP, as compared to the remaining five round-bellied horses, was also notable. The C_tot_ values obtained in the study are likely not true physiologic values because they also include compliance of the breathing system. However, a between-group comparison is still relevant since the same exact equipment was used in all instances. 

In the current study, the 15° RTP was associated with an apparent greater negative cardiovascular effect compared to HP. With the caveat that the study was not designed to test the cardiovascular effects of 15° RTP and these data were not compared statistically, our findings appear to be in contrast with reports using smaller RTP angles in horses [13,14] and steers [30] as well greater angles in humans [27,28,40]. While positive pressure ventilation and the constant-dose isoflurane likely had some contribution to the hypotension observed in the current study, the difference between RTP and HP can be attributed to the 15° RTP given the study design with each horse receiving both treatments and thus serving as their own control. Dobutamine and phenylephrine were used as pharmacologic blood pressure support. Dobutamine is the preferred drug to treat hypotension in anesthetized horses [41] although it can cause sinus tachycardia if administered at rates greater than 3 to 5 μg/kg/min or in patients with inadequate intravascular volume [41,42,43]. Accordingly, sinus tachycardia associated with increasing infusion rates of dobutamine occurred in several horses of the current study, especially during RTP, which became a limiting factor for the administration of dobutamine. It is unlikely that the healthy horses of the study had inadequate intravascular volume. Instead, increases in regional vascular capacitance associated with the 15° RTP, as shown in humans [44,45], likely decreased preload and emulated a decreased intravascular volume. Thus, phenylephrine was employed for vasoconstriction [46] and its requirement was markedly greater during RTP than HP. The study suggests that the effect of different RTP angles on cardiovascular function in anesthetized horses, including the physiological basis and optimum therapeutic strategies, merits further investigation since hypotension is a significant risk factor for anesthetic morbidity and mortality in horses [8].

Isoflurane causes dose-dependent decreases in MAP and skeletal muscle blood flow [43]. In the current study, the isoflurane dose was standardized at 1.2 × MAC to ensure the same intra-individual anesthetic depth for HP and RTP and to minimize the confounding effects of additional drugs possibly required to correct insufficient anesthetic depth. However, this approach could have resulted in different inter-individual anesthetic depths, possibly excessive in some cases given the absence of invasive procedures. The cardiovascular support with dobutamine and phenylephrine was effective in correcting hypotension. Inadequate blood flow to skeletal muscle, if it occurred, was not sufficient to result in clinical signs of post-anesthetic myopathy although subclinical myopathy cannot be ruled out since muscle enzymes were not determined. Phenylephrine does not change skeletal muscle blood flow in anesthetized horses, but dobutamine can be effective in mitigating anesthetic-induced skeletal muscle hypoperfusion and post-anesthetic myopathy [42,43,47,48]. In horizontally positioned isoflurane-anesthetized horses, phenylephrine significantly decreased intestinal blood flow, most profoundly at the colon [49]. Studies are needed to characterize the intestinal regional arterial and venous responses to the 15° RTP and the effects of phenylephrine in anesthetized horses.

The junctional escape-capture bigeminy seen in one of the horses in the current study has not been previously reported in horses. Only two reports could be found in the veterinary literature, one in a domestic shorthair cat [50] and another in a Yorkshire Terrier dog [51]. In this horse, the junctional escape-capture bigeminy was not related to RTP since it occurred during HP. It coincided with starting dobutamine to correct hypotension in the presence of sinus bradycardia (heart rate of 20–25 beats/min). Upon starting dobutamine, occasional junctional escape beats were observed, followed by a junctional rhythm and then junctional escape-capture bigeminy. Bradycardia was the common finding among the two reported veterinary cases [50,51] and in this horse. In this horse, the escape-capture bigeminy subsided as the heart rate increased during anesthesia. This did not happen in the second anesthetic episode (RTP) in which bradycardia did not occur. In escape-capture bigeminy, a conducted complex can only follow an escape complex if the R-P interval of the retrograde P wave is sufficiently long (i.e., via the slow atrioventricular nodal pathway) to be conducted back to the ventricles via the fast atrioventricular nodal pathway. When the P wave is caused by retrograde conduction via the fast atrioventricular nodal pathway, anterograde atrioventricular nodal conduction is blocked, resulting in a blocked P wave [52], which was occasionally evident in the horse of the study. 

The study has several limitations. First, the exclusion of one horse from statistical comparisons resulted in a relatively small sample size. However, it also resulted in a very homogeneous group of horses regarding body shape (all round-bellied) and thoracic dimensions, which may have increased the chances of finding statistically significant differences. Despite the relatively small sample size, statistical analysis indicated that the treatment effect explained approximately half of the total variance, with a very small chance (<1%) of randomly obtaining the observed effect. It should be recognized nonetheless that our results are conditioned to a small homogeneous group of horses, which likely does not fully represent the wider equine population. Larger studies are needed to confirm or refute our findings. The use of xylazine and dexmedetomidine for pre-anesthetic medication could be considered another limitation. However, these α-2 adrenergic receptor agonists were used at approximately equipotent sedative doses, and the available literature suggests that there are no significant cardiopulmonary differences between them during isoflurane anesthesia [53] or total intravenous anesthesia with ketamine and midazolam [54] in horses. Another potential concern was the estimation of venous admixture via calculation of F-shunt instead of deriving it from a calculation based on mixed venous, capillary, and arterial oxygen contents, using the traditional Berggren shunt equation [55]. However, venous admixture can be accurately estimated by calculating F-shunt, as demonstrated during one- and two-lung ventilation in anesthetized sheep [35]. This approach has been utilized in anesthetized horses in several publications [13,14,36]. The arteriovenous oxygen content difference used in the F-shunt calculations originated from mechanically ventilated humans [37] but is similar to that of isoflurane-anesthetized, dorsally recumbent horses [23,38]. Further, the F-shunt strongly correlated with shunt fraction estimated by the Berggren shunt equation during different infusion rates of dobutamine in isoflurane-anesthetized horses [56]. The markedly higher phenylephrine requirement during RTP than HP could be considered another concern but research in isoflurane-anesthetized horses showed that phenylephrine did not affect PaO_2_ in horses positioned in HP [49]. Finally, the use of a point-of-care (POC) instrument to determine arterial blood gas tensions could be criticized. However, the instrument was validated against our laboratory’s reference values, is routinely verified for quality control, and all samples were analyzed immediately after collection using the same POC instrument. In addition, the POC instrument used in our study was previously found to have good agreement with a central analyzer [57]. 

## 5. Conclusions

Under the conditions of this study, a 15° RTP resulted in better oxygenation than HP in dorsally recumbent, isoflurane-anesthetized horses, although worsening of oxygenation may occur in individual horses. Further studies are warranted to determine the effect of RTP in horses with different body conformation (i.e., round-bellied vs. flat-bellied horses) and to characterize the cardiovascular responses to different RTP angles before it can be recommended for clinical practice.

## Figures and Tables

**Table 1:** Demographic information of horses used in the study to determine the effect of a 15° reverse Trendelenburg position on arterial oxygenation during isoflurane anesthesia and dorsal recumbency.

Variable	Horse					
	#1	#2	#3	#4	#5*	#6
Breed	QH	Arabian	Mixed	QH	Mixed	QH
Sex	Gelding	Gelding	Gelding	Gelding	Gelding	Female
Age (years)	9	10	5	6	13	8
Body weight (kg)	536	397	417	467	484	471
Body shape	RB	RB	RB	RB	FB	RB
Thoracic depth (cm)	75	74	67	70	72	72
Thoracic width (cm)	34	27	30	31	34	36
Thoracic length (cm)	99	93	91	95	141	93
Thoracic circumference (cm)	192	172	171	181	184	188

For breed, QH = Quarter Horse; For body shape, RB = Round-bellied and FB = Flat-bellied. Thoracic dimensions were obtained as previously described [12]. Depth = vertical distance between dorsal spinous processes and sternum; Width = horizontal depth between points of shoulder (scapulohumeral joint); Length = diagonal from point of shoulder to distal end of last rib; Circumference = caudal to withers. * = Horse #5 was considered an outlier based on body shape and thoracic length.

**Table 2:** Mean ± SD arterial partial pressures of oxygen (PaO_2_), carbon dioxide (PaCO_2_), end-tidal carbon dioxide (P_ET_CO_2_), arterial-to-inspired oxygen ratio (P–F ratio), alveolar dead-space ventilation (Vd/Vt), and oxygen content-based index (F–shunt) in isoflurane-anesthetized, dorsally recumbent, mechanically ventilated horses (*n* = 5) positioned either horizontally (HP) or in a 15° reverse Trendelenburg position (RTP). An asterisk indicates a significant difference (*p* < 0.05) between treatment for each variable. Within treatment, differences are indicated with superscript letters (time points without a common letter are significantly different). Variables without notation were not significantly different.

Variable	Position	Minutes of Anesthesia				Overall Mean ± SD (95% CI)
		30	60	90	120	
PaO_2_ (mmHg)	HP	415 ± 21	449 ± 32	413 ± 27	420 ± 33	424 ± 17 (398, 451)
	RTP	484 ± 53	486 ± 79	497 ± 60 *	502 ± 52 *	504 ± 14 (482, 525)
P–F ratio (mmHg)	HP	432 ± 25	466 ± 35	427 ± 27	433 ± 34	440 ± 18 (411, 468)
	RTP	510 ± 56 *	505 ± 81	513 ± 62 *	518 ± 53 *	512 ± 5 (503, 520)
F–shunt (%)	HP	15 ± 1	13 ± 2	15 ± 1	15 ± 2	15 ± 1 (13, 17)
	RTP	10 ± 3 *	10 ± 6	10 ± 4 *	9 ± 3 *	10 ± 0.3 (9, 10)
PaCO_2_ (mmHg)	HP	48 ± 7	53 ± 4	52 ± 6	51 ± 10	52 ± 2 (49, 55)
	RTP	49 ± 4	54 ± 7	54 ± 5	57 ± 6	53 ± 2 (49, 57)
P_ET_CO_2_ (mmHg)	HP	35 ± 2	38 ± 2	36 ± 4	35 ± 6	36 ± 1 (34, 38)
	RTP	38 ± 3	39 ± 4	38 ± 3	37 ± 3	38 ± 1 (36, 39)
Vd/Vt (%)	HP	26 ± 8	29 ± 2	31 ± 3	32 ± 6	29 ± 3 (25, 33)
	RTP	24 ± 2 ^a^	28 ± 3 ^ab^	30 ± 4 ^b^	35 ± 4 ^b^	29 ± 5 (22, 36)

**Table 3:** Median (range) values of rectal temperature (RT) and doses of dobutamine and phenylephrine in isoflurane-anesthetized horses (*n* = 6) positioned either horizontally (HP) or in 15° reverse Trendelenburg position (RTP). Body position (HP or RTP) during the first anesthetic event is also indicated.

Variable	Position	Horse						Overall
		#1	#2	#3	#4	#5	#6	
RT (°C)	HP	36.0 (35–36.6)	36.4 (36.1–37.3)	36.5 (36.3–36.6)	36.3 (36.0–36.8)	36.3 (35.3–37.2)	37.8 (36.6–37.8)	36.4 (35.6–37.1)
	RTP	37.2 (36.2–37.2)	36.3 (36.2–37.4)	37.2 (36.2–37.4)	36.4 (36.3–36.9)	35.0 (34.2–36.6)	36.7 (36.7–37.1)	36.4 (36.0–36.9)
Dobutamine (μg/kg/min)	HP	1.4	2.1	1.5	1.7	0.5	1.2	1.5
	RTP	2.1	4.8	1.5	1.1	3	1.8	1.8
Phenylephrine (μg/kg)	HP	5.6	0	6.2	0.9	0	0	0.9
	RTP	42.9	8.1	2.4	28.9	12.4	39.9	29
Body position during first anesthesia	-	HP	RTP	RTP	HP	RTP	HP	-

## Data Availability

The data set used for statistical analysis is available upon reasonable request.
